# Secondary Metabolites of *Lasiodiplodia theobromae*: Distribution, Chemical Diversity, Bioactivity, and Implications of Their Occurrence

**DOI:** 10.3390/toxins12070457

**Published:** 2020-07-17

**Authors:** Maria Michela Salvatore, Artur Alves, Anna Andolfi

**Affiliations:** 1 Department of Chemical Sciences, University of Naples ‘Federico II’, 80126 Naples, Italy; mariamichela.salvatore@unina.it; 2 CESAM-Centre for Environmental and Marine Studies, Department of Biology, University of Aveiro, 3810-193 Aveiro, Portugal; artur.alves@ua.pt; 3 BAT Center-Interuniversity Center for Studies on Bioinspired Agro-Environmental Technology, University of Naples ‘Federico II’, 80126 Naples, Italy

**Keywords:** *Botryodiplodia theobromae*, *Botryosphaeria rhodina*, natural products, bioactivity

## Abstract

*Lasiodiplodia theobromae* is a plant pathogenic fungus from the family Botryosphaeriaceae that is commonly found in tropical and subtropical regions. It has been associated with many hosts, causing diverse diseases and being responsible for serious damages on economically important crops. A diverse array of bioactive low molecular weight compounds has been described as being produced by *L. theobromae* cultures. In this review, the existing literature on secondary metabolites of *L. theobromae*, their bioactivity, and the implications of their occurrence are compiled. Moreover, the effects of abiotic factors (e.g., temperature, nutrient availability) on secondary metabolites production are highlighted, and possible avenues for future research are presented. Currently, a total of 134 chemically defined compounds belonging to the classes of secondary metabolites and fatty acids have been reported from over 30 *L. theobromae* isolates. Compounds reported include cyclohexenes and cyclohexenones, indoles, jasmonates, lactones, melleins, phenols, and others. Most of the existing bioactivity studies of *L. theobromae* metabolites have assessed their potential phytotoxic, cytotoxic, and antimicrobial activities. In fact, its host adaptability and its ability to cause diseases in plants as well as in humans may be related to the capacity to produce bioactive compounds directly involved in host–fungus interactions.

## 1. Introduction

*Lasiodiplodia theobromae* (=*Botryodiplodia theobromae*), whose sexual morph has been classified as *Botryosphaeria rhodina*, is an ascomycete fungus that belongs in the class Dothideomycetes, order Botryospheriales, and family Botryosphaeriaceae [1]. Geographically, it can be found almost everywhere around the world, but with particular incidence in tropical and subtropical regions, being one of the most commonly found species of Botryosphaeriaceae [1,2]. Similar to most species in the family Botryosphaeriaceae, it is a non-host specific plant pathogen or endophyte, occurring on diverse crops and trees where it has been associated with diseases such as fruit rot, root rot, dieback, and canker [3,4]. In fact, *L. theobromae* has been associated with more than 500 different plant hosts [3,5]. Additionally, it has been occasionally documented to be the causal agent of infections in humans [6] with a variety of infections reported in both immunocompetent and immunocompromised patients including sinusitis, keratitis, pneumonia, and cutaneous lesions [7,8,9].

With the introduction of DNA sequence analysis into the delimitation of species in *Lasiodiplodia*, it became apparent that *L. theobromae* represented a complex of cryptic species [10]. Thus, any reports concerning *L. theobromae* that predate the use of DNA sequencing for the identification of isolates must be considered with caution, as it is possible that some may in fact refer to closely related cryptic species.

Apart from its importance as a pathogen, *Lasiodiplodia theobromae* has caught the attention of researchers due to its ability to produce biotechnologically relevant compounds, ranging from enzymes [11] to polysaccharides [12] and secondary metabolites [13]. In recent years, the number of reports on secondary metabolites produced by *L. theobromae* has increased considerably [11,13,14,15,16,17,18,19]. In this review, we compile the available data on the multiplicity of secondary metabolites that are known to be produced by this fungus, particularly focusing on low molecular weight compounds. In addition, when available, information concerning their biological activities and potential applications is presented.

## 2. Secondary Metabolites

*Lasiodiplodia theobromae* produces a plethora of low molecular weight compounds with original structures and bioactivities. In this respect, this fungus produces metabolites with huge structural diversity belonging to different classes of natural products, including diketopiperazines, jasmonates, lactones, melleins, and others (Table 1).

This structural variability also explains their broad spectrum of activities and functions. Of great interest is the role of secondary metabolites produced by *L. theobromae* during the interaction with other organisms. In fact, secondary metabolites could be produced as a physiological response to multiple biotic and abiotic stimuli. *L. theobromae* has a vast number of hosts with which it establishes symbiotic relationships. Particularly interesting is the symbiotic association with plants that includes both endophytic and pathogenic interactions [20]. In fact, when *L. theobromae* grows inside living plant tissues as an endophyte, secondary metabolites may fulfill very different functions, such as mediating communication, nutrient acquisition, and defense. Meanwhile, in pathogenic interactions, secondary metabolites play crucial rules as virulence factors, causing specific symptoms observed when *L. theobromae* infects plants and humans [21].

In this review, the wide variety of studied *L. theobromae* strains allows evaluating the differences in metabolite production that might be related to different hosts, as well as to the symbiotic association established between a fungus and host (Table 2). In order to better understand the role of secondary metabolites in the virulence of *L. theobromae*, investigations were conducted testing in vitro the effect of abiotic factors such as temperature [13,19] and nutrient availability [22] on the extrolites production. Moreover, studies have compared secondary metabolites produced by *L. theobromae* in liquid medium with the ones obtained after an incubation period of the fungus in the host tissue, and metabolites detected in this latter condition were named vivotoxins by He and co-workers [23].

### 2.1. Cyclohexenes and Cyclohexenones

**Figure 1:**
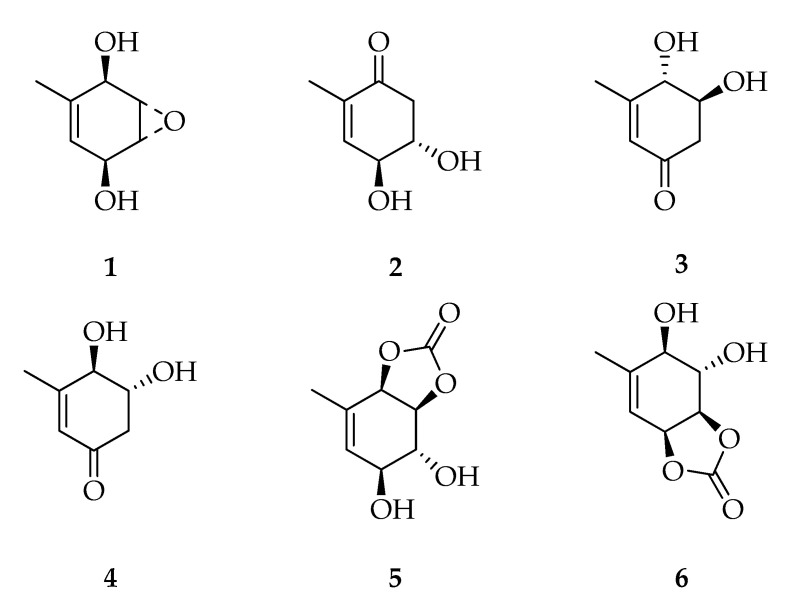
Structures of cyclohexenes and cyclohexenones.

Theobroxide (**1**) is the founder product of the class of “cyclohexenes and cyclohexenones” (Figure 1). It is a polyketide with a cyclohexendiole epoxide subunit isolated for the first time from *L. theobromae* IFO31059 [30]. Subsequently, three dihydroxyhexanones (**2**–**4**) and two carbonyldioxy derivatives (**5**,**6**) of theobroxide were isolated from cultures of *L. theobromae* [38,40]. A nucleophilic reaction of the *trans*-diaxial HCO_3_^-^ ion could be responsible for the formation of several theobroxide analogues, and based on the stereochemical aspects, it can be speculated that this reaction is a non-enzymatic mechanism. The first studies on this class of compounds were aimed to identify new potato microtuber substances. In fact, **1** strongly stimulated microtuber formation and enhanced the inductive effect of jasmonic acid when they were used in combination, in both old and new tissue of potato [52]. Moreover, **1** increases lipoxygenase (LOX) activity during potato tuber formation in vivo [53] and in vitro [52]. These findings might be linked to the capacity of **1** to induce the jasmonic acid synthesis pathway, and this compound is able to induce defense response against external stressors and from pathogen attack in plants. In this respect, **1** inhibits disease development by inducing defense response in *Nicotiana benthamiana* [54]. Theobroxide is also capable of promoting the flower-bud formation in other plants, such as morning glory (*Pharbitis nil*) [55]. 

### 2.2. Depsidones

**Figure 2:**
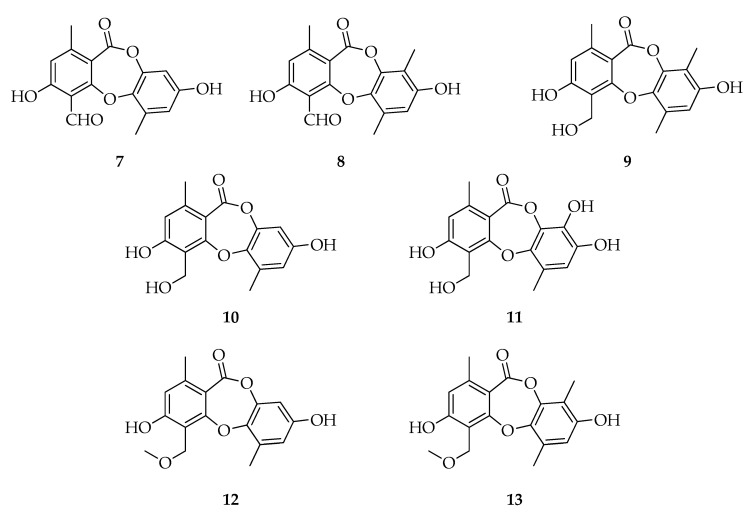
Structures of depsidones.

Depsidones are ester-like depsides, or cyclic ethers, which are related to the diphenyl ethers and synthesized through the polymalonate pathway. Their structure is based on a 11H-dibenzo[b,e]dioxepin-11-one ring system where bridging is at the phenolic group in the p-position (Figure 2). Several biological activities were reported for compounds belonging to the depsidones family, such as antiproliferative, antimalarial, cytotoxic, antibacterial, radical scavenging, antihypertensive, anti-trypanosomal anti-malarial, anti-leishmanial, herbicidal, larvicidal, aromatase and cholinesterase inhibitor, antioxidant, and antifungal [56,57].

A strain of *Botryosphaeria rhodina* isolated from stems of *Bidens poilosa* (Asteraceae) was reported as a producer of botryorhodines A, B, C, D (**7**–**10**) [42]. Subsequently, **7** and **8** were detected in culture of *L. theobromae* M4.2-2, together with the new botryorhodine I (**11**) and several analogues, such as 1H-dibenzo[b,e] [1,4]dioxepin-11-one,3,8-dihydroxy-4-(methoxymethyl)-1,6-dimethyl (**12**) and simplicildone A (**13**). This strain also produced (3*R*)-de-*O*-methyllasiodiplodin (**62**), (*R*)-nordinone (**75**), and (*R*)-mellein (**81**) [51] (Figure 2).

The antifungal activities of **7**–**10** were tested against pathogenic fungi, such as *Aspergillus terreus* and *Fusarium oxysporium*. In particular, agar diffusion assays showed that **7** and **8** are active and their minimal inhibitory concentrations (MICs) are, respectively, 26.03 and 49.70 µ against *A. terreus* and 191.60 and 238.80 µM against *F. oxysporium*. Compounds **9** and **10** turn out to be not active in the agar diffusion assays, and these results may be related to the lack of aldehydic group in their structures. Furthermore, **7** and **8** show from moderate to weak cytotoxic activities against HeLa cell lines [42]. Compounds isolated by Umeokoli and co-workers [51] were examined for their antiproliferative potential against the mouse lymphoma cell line L5178Y. Only **12** exhibited a potent cytotoxic activity with an IC50 value of 7.3 25 mM. In addition, all compounds were assessed for their antibacterial activities against a panel of Gram-positive and Gram-negative bacterial strains, but no depsidones showed significant activity against the tested organisms at a dose of 64 µg mL^−1^.

### 2.3. Diketopiperazines

**Figure 3:**
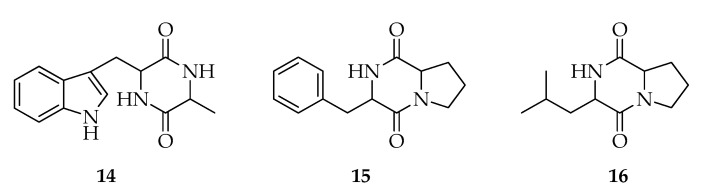
Structures of diketopiperazines.

Diketopiperazines are a class of natural products represented by cyclic dipeptides obtained by the condensation of two α-amino acids (Figure 3). These compounds are receiving increased interest for their remarkable bioactivities [58,59]. The *Lasiodiplodia* species have shown the production of diketopiperazines and derivatives. In particular, *cyclo*-(Trp-Ala) (**14**) and six sulfur-containing diketopiperazines were identified from cultures of the endophyte *L. pseudotheobromae* F2 [60]. *Cyclo*-(Trp-Ala) was also produced by *L. theobromae* strains from humans and the coconut tree. A comparative study that tested its production at two different growth temperatures showed that **14** is exclusively produced when the fungus is incubated at 37 °C [19]. In the same study [19], **14** was tested for cytotoxicity on mammalian cells, showing a weak activity. *Cyclo*-(Phe-Pro) (**15**) and *cyclo*-(Leu-Pro) (**16**) were produced by a strain of *L. theobromae* isolated from *Solanum nigrum* [48]. 

### 2.4. Indoles

**Figure 4:**
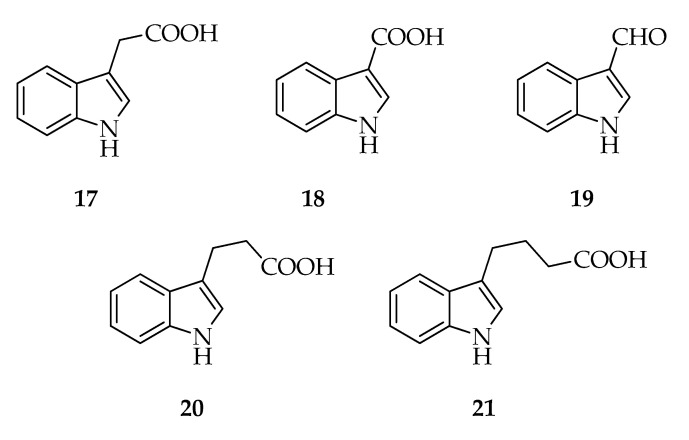
Structures of indoles.

Auxin is the most important plant hormone regulating almost all aspects of plant growth and development. Indole-3-acetic acid (**17**) is the most studied auxin in plants, and its biosynthesis pathway has been investigated for over 70 years. Indole-3-acetic acid can be *de novo* synthesized via tryptophan-dependent pathways or tryptophan-independent pathways [61]. Several fungal species produce **17**, such as *Ascochyta pisi*, *Giberella fujikuroi*, *Pyricularia oryzae*, and *Rhizoctonia* species [62]. 3-indolecarboxylic acid (3-ICA, **18**) has been reported as *L. theobromae* metabolite, and its biosynthesis in microorganisms might start from l-tryptophan via **17** [63] or, as observed in several *Orobanche* species (holoparasitic dicotyledonous plants), via 3-indolcarbaldehyde (**19**) [64]. This latter hypothesis is supported by the presence of some biosynthetic intermediates in the fungal metabolism. In fact, **18** and **19** have been detected by GC-MS in cultures of an unidentified endophytic strain of Lasiodiplodia, which is closely related to *L. pseudotheobromae*, but **17** has not been reported [14]. Furthermore, both **18** and **19** were already documented as being produced by *L. theobromae* (Figure 4).

The biological role of 3-indolecarboxylic acid has long been neglected, but some studies have highlighted its potential role in plant as phytoalexin. In fact, **18** has been identified as a mediator of induced resistance in Arabidopsis against plant pathogens [65].

Interestingly, for the first time in culture organic extracts of *L. theobromae*, several phytohormones, including 3-indoleacetic acid (**17**), indole-3-propionic acid (**20**), and indole-3-butyric acid (**21**) were identified by liquid chromatography-electrospray tandem mass spectrometry. Indole-3-propionic acid might be synthesized from the reduction of indole-3-pyruvic acid, while in some species, such as corn and *Arabidopsis thaliana*, **21** is synthesized from the elongation of the **17** chain by adding a unit of acetyl coenzyme A, as happens in the biosynthesis of fatty acids (Figure 4). Furthermore, the conversion of **21** in **17** was also observed [66]. 

The concentrations of three indoles (**17**, **20**, and **21**) in cultures of 2334, 1517, and 83 strains of *L. theobromae* were reported to be in the range 0.0407–0.0066 µg mL^−1^, which is much less than the jasmonic acid (JA, **22**) concentration obtained in the same cultures [44]. 

3-Indolecarboxylic acid produced by human (CBS339.90) and coconut tree (CAA019) strains of *L. theobromae* was tested for phytotoxicity and cytotoxicity. Compound **18** is toxic for tomato leaves, causing a lesion of 2.3 mm after 10 days exposure. Furthermore, **18** showed toxicity for mammalian cell lines (Vero and 3T3 cells), especially to 3T3 cells, inducing 100% cell mortality when the metabolite concentration is 1 mg mL^−1^ [19].

### 2.5. Jasmonates 

**Figure 5:**
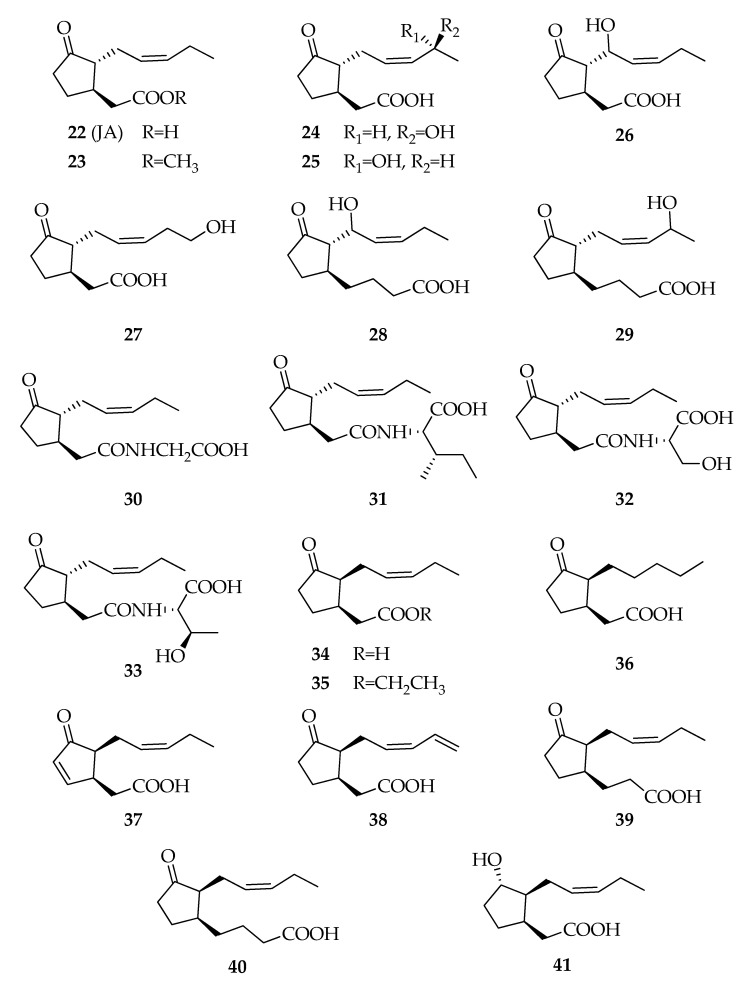
Structures of jasmonates.

Jasmonates are a class of natural compounds structurally related to jasmonic acid (JA, **22**). The founder product of this compounds series is a well-known phytohormone produced by plants in response to phytophagous, necrotrophic microbes, and abiotic stressors [67,68,69]. JA is one of the most important signal molecules in the plant defense response against pathogens in addition to salicylic acid (SA). Through signal transduction using these molecules, plants respond to pathogen attack or external stresses by rapid changes in gene expression, resulting in the induction of genes involved in the defense response, such as the pathogenesis-related (PR) proteins. Therefore, the genes of PR proteins are induced and accumulated in host plants as a result of pathogen infection or abiotic stresses. Furthermore, jasmonic acid is involved in several physiologic processes including seed germination, blooming period, and senescence [70]. Several *Lasiodiplodia* species, in particular *L. theobromae*, with different lifestyles (i.e., pathogens, endophytes) and associated to diverse hosts, turned out to be in vitro producers of **22** and analogues (Figure 5). Jasmonic acid is not exclusively produced by *Lasiodiplodia* species; it is also produced by many fungal species from the genera *Aspergillus*, *Collybia*, *Coprinus*, *Fusarium,* and *Gibberella* [44]. Compound **22** is synthesized from lipid-constituents via one of the branches of the lipoxygenase (LOX) pathway [71]. Interestingly, *L. theobromae* efficiently oxidizes linolenic and linoleic acids (C18:3n3 and C18:2n6) sequentially to 9*R*-hydroperoxides and to unstable allene oxides, which are possible precursors of **22** [72]. Jasmonic acid was for the first time isolated in 1971, along with its already known methyl esters, from a culture of a *L. theobromae* strain [25]. Some *Lasiodiplodia* species produce several jasmonates obtained from the esterification of **22** with other compounds, such as lasiojasmonates A–C by *Lasiodiplodia* sp. isolated from declining grapevine plants [73], which was subsequently identified as a new species named *Lasiodiplodia mediterranea* [74].

Among the compounds structurally related to **22**, compounds with propanoic and butanoic acid instead of acetic acid, 7-*iso*-jasmonic acid (**34**) and its analogues (e.g., dehydro–, dihydro–, and hydroxylated derivatives) have been reported as products of *L. theobromae* from *Cuban oranges* [26,27,28]. *N*-Jasmonyls conjugated with glycine, serine, isoleucine, and threonine (**30–33**) have been isolated from cultures of a Brazilian strain associated to *Citrus sinensis* [44].

The investigations on bioactive properties of jasmonates are essentially focused on the potential role of these metabolites in the interaction between host and pathogen. As expected, jasmonic acid is capable to induce the formation of microtuber in potato [29], but it is also phytotoxic for rose [29], tomato [19], and grapevine [73]. The most relevant results were obtained from leaf puncture assays performed on different plants. In fact, 1 mg ml^−1^ solutions of **22** showed toxicity on the non-host plant tomato (lesion size 4.7 mm), on cork oak (lesion area 11.96 mm^2^), and on grapevine (lesion area 7.04 mm^2^). Conversely, jasmonic acid esters showed no phytotoxicity [73]; hence, it can be deduced that the carboxylic group is involved in the biological activity of **22**.

### 2.6. Lactones and Analogues

**Figure 6:**
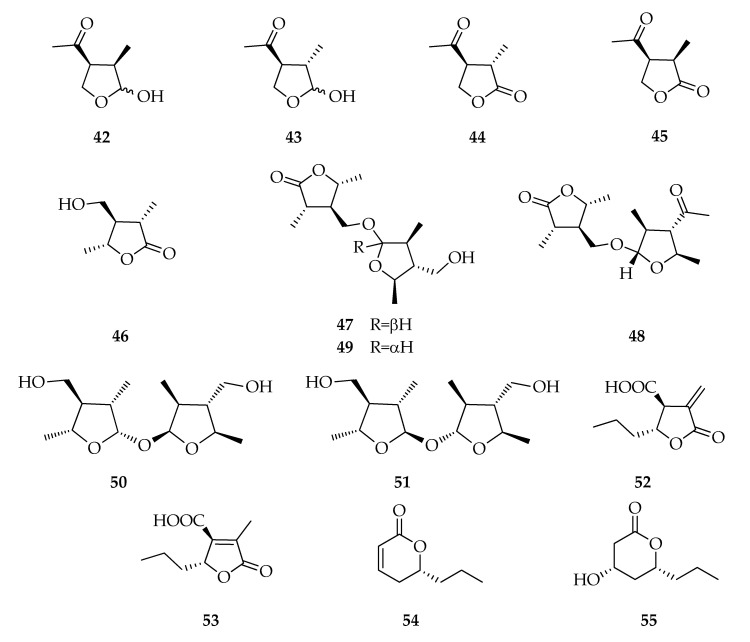
Structures of lactones and analogues.

Furano-2-ones, furanoles, and pyran-2-ones are often isolated from *L. theobromae* cultures (Figure 6). (3*R*,4*S*)-(-)-Botryodiplodin (**42**) was the first compound, belonging to this series, isolated from a liquid culture of a ligninolytic strain of *L. theobromae*, and reported by [24] as a new antibiotic product, but its structure and stereostructure were subsequently determined [75,76,77,78]. Moreover, (±)-botryodiplodin and its stereoisomers were also prepared through numerous synthetic procedures [79]. (3*R*,4*S*)-(-)-Botryodiplodin was also isolated as a product of other fungal species, such as *L. mediterranea* [73] and *M. phaseolina* [80]. This compound is a hemiacetal in equilibrium between two epimeric forms on the anomeric carbon, and recently, the epimer of **42**, named (3*S*,4*S*)-3-*epi*-botryodiplodin (**43**), was identified for the first time as natural product by Félix and co-workers [13], who isolated (**43**) by cultures of grapevine strains of *L. theobromae*. Interestingly, oxidation products on C-2 of botryodiplodins (**44**,**45**) were isolated from strains of *L. theobromae* associated with different hosts, in particular coconut tree, human, and grapevine [13,19].

(3*S*,4*R*,5*R*)-4-Hydroxymethyl-3,5-dimethyldihydro-2-furanone (**46**) was isolated for the first time as a natural product from the endophytic strain PSU-M35 of *L. theobromae* together with its esters namely botryosphaerilactones A-C (**47**–**49**) [41], but afterwards, **46** was also isolated from other strains (Table 2). 

Lasiolactols (**50**,**51**), two dimeric γ-lactols, are acetalic forms of furanols produced by grapevine strains of *L. theobromae*, but they are already known from *L. mediterranea* associated with grapevine decline in Sicily, Italy [81]. 

A strain of *L. theobromae* isolated from rotted mango branches is the first producer of (3*S*,4*R*)-3-carboxy-2-methylene-heptan-4-olide (**52**), together with its well-known isomer decumbic acid (**53**). Furthermore, **52** is also considered a *vivotoxin* because it was isolated from bananas incubated with the fungal mycelium [23]. 

(*R*)-2-Octeno-δ-lactone, also called lasiolactone (**54**), and tetrahydro-4-hydroxy-6-propylpyran-2-one (**55**) are two pyran–2–ones isolated from the strain PSU–M114 [38], but **54** was also detected in a culture of a coconut tree strain of *L. theobromae* [31] (Figure 6).

Concerning the biological activities of this compounds series, (3*R*,4*S*)-(-)-botryodiplodin is a natural mycotoxin with a variety of biological activities, such as anticancer, antibacterial, antifungal, phytotoxic, mitogenic, and antifertility activities [79], and it may play a role in plant diseases [82]. Although **42** showed no activity in leaf puncture assays on tomato, grapevine, and cork oak leaves at 1 mg mL^−1^ [13,73], its acetate derivative and (3*S*,4*S*)-3-*epi*-botryodiplodin caused lesions with diameters of 8.7 and 7.0, respectively [13]. Even in the case of furanones (**44**,**45**), the biological activity is affected by stereochemistry. In fact, (3*R*,4*S*)-4-acetyl-3-methyl-dihydro-furan-2-one (**45**) produced necrosis on tomato leaves (5.0 mm), but its epimer (**44**) was not phytotoxic. The lactone caused lesions of 6.0 mm, while its acetate derivative and lasiolactols turned out not to be phytotoxic on tomato leaves [13].

In cytotoxicity tests conducted on mammalian cells (i.e., Vero and 3T3 cell lines), (**43**) was more toxic than **42** on both cell lines, especially on 3T3 cells. In fact, **43** at a concentration of 0.5 mg mL^−1^ caused 100% of 3T3 cell mortality. (3*R*,4*S*)-(-)-Botryodiplodin and its acetate derivative had the same cytotoxicity to 3T3. Conversely, **42** was more toxic than botryodiplodin acetate to Vero cells. Furthermore, **44** and **45** were able to reduce the cell viability at about 5% to 3T3 cells. (3*S*,4*R*,5*R*)-4-hydroxymethyl-3,5-dimethyldihydro-2-furanone (**46**) caused 100% of 3T3 cell death (1 mg mL^−1^), and the same activity was found for its acetate derivatives [13]. 

### 2.7. Lasiodiplodins

**Figure 7:**
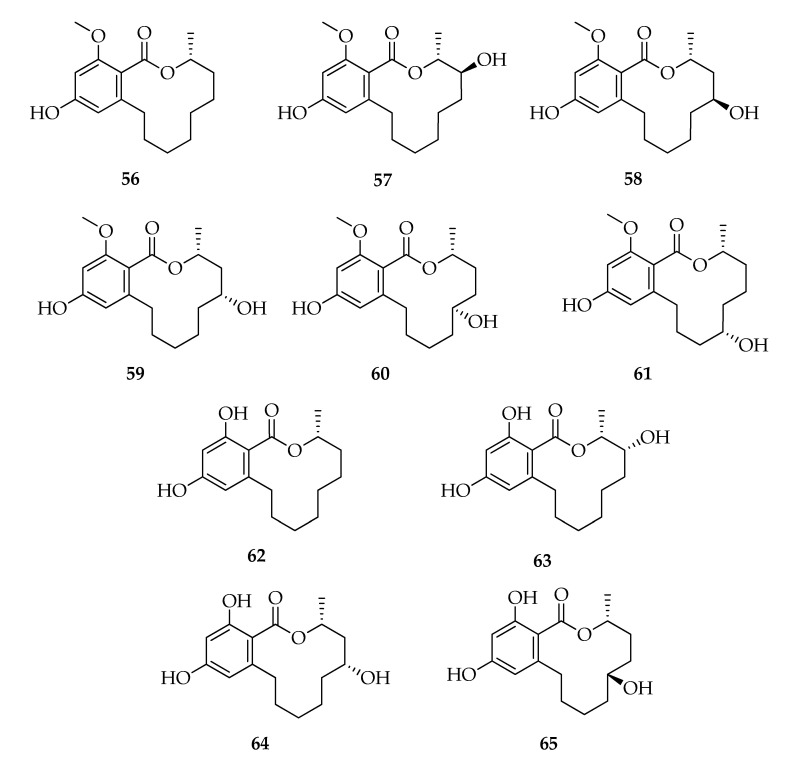
Structures of lasiodiplodins.

**Figure 8:**
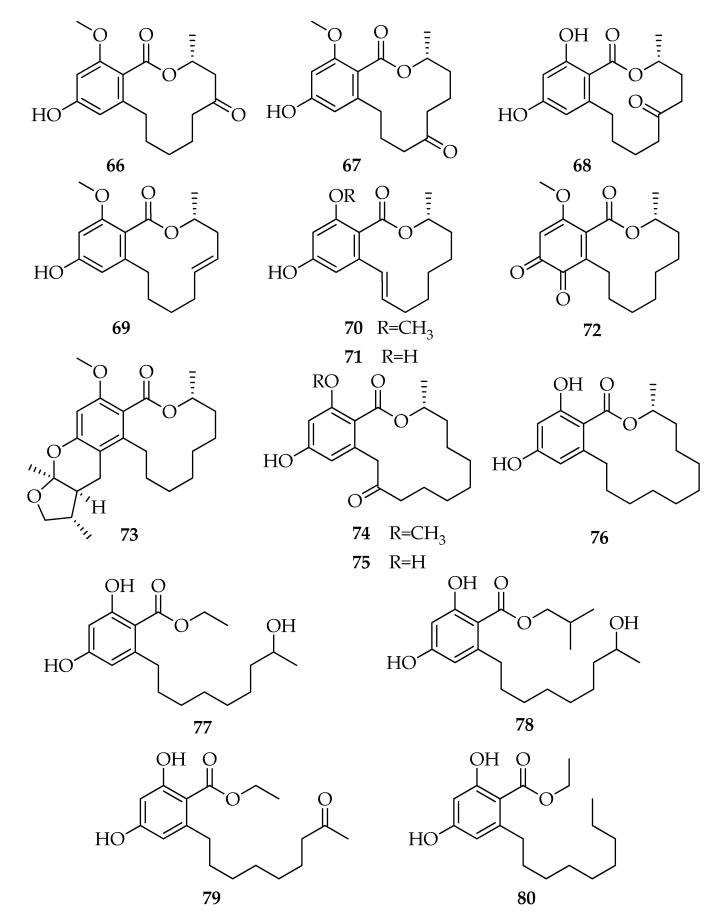
Structures of lasiodiplodins.

Lasiodiplodins are 12-membered benzenediol lactones, octaketides possessing a resorcinol aromatic ring and a macrocyclic lactone [83] (Figure 7). The founder compound of this class of natural products is (3*R*)-lasiodiplodin (**56**), which was isolated from a *L. theobromae* strain together with its (3*R*)-de-*O*-methyl-analogue (**62**) [25]. Subsequently, **56** and its analogues were reported as products of several strains of *L. theobromae*. The most significant structural modifications observed in **56** and **62** are in the hydroxyl groups on the lactone ring (from C-4 to C-7) and in the stereochemistry [i.e., (3*R*,4*S*)-4-hydroxy-lasiodiplodin (**57**) or (3*R*,4*R*)-4-hydroxy-de-O-methyl-lasiodiplodin (**63**)] or in the presence of carbonylic group [i.e., (3*R*)-5-oxo-lasiodiplodin (**66**) or (3*R*)-6-oxo-de-*O*-methyl-lasiodiplodin (**68**)] (Figure 7). Recently, some dehydroderivatives, such as (3*R*,9*E*)-9-etheno-de-*O*-methyl-lasiodiplodin (**71**), were found in cultures of *L. theobroamae* (Table 2). Moreover, lasiodiplodins with wide structural modifications in the resorcinol and lactone rings from several strains of *L. theobromae* have been reported (Figure 8). The resorcinol ring oxidation lead to the ortho quinone formation or a furo-pyran moiety as reported for **72** and **73**, respectively, while lactone ring modifications occur in (*R*)-zearelenone (**76**) and *epi*-8,9-dihydrogreensporone C (**74**) becoming 14-membered benzenediol lactones (Figure 8). The macrocyclic ring can be affected by modifications that cause the opening of the ring and diverse substituents can be in ortho to the carboxylic group, such as hydroxyheptyl or hydroxynonyl moieties (**77–80**). Interestingly, several well-known and new lasiodiplodins (Table 2) were isolated from a non-identified endophytic strain of *L. theobromae* isolated from a brown alga from the South China sea [36]. Even in cultures of the endophytic strain 318# of *Lasiodiplodia* sp. isolated from *Excoecaria agallocha* were identified several compounds belonging to the lasiodiplodins series [18,47]. Although lasiodiplodins production has been reported in other fungal species (i.e., *Sarocladium kiliense*, *Syncephalastrum racemosum*) [84,85] and in many plants (i.e., *Ampelopsis japonica, Euphorbia splendens, Macroptilium lathyroides*) [83,86], these compounds are frequently identified in cultures of *Lasiodiplodia theobromae* (Table 2). Furthermore, several authors assumed that lasiodiplodins may not be plant metabolites but may be produced by symbiotic fungi [87]. Chemotaxonomy studies demonstrated that secondary metabolites have been used successfully in fungal taxonomy [88], and lasiodiplodins may be used as potential chemotaxonomic markers. In this respect, the exclusive production of lasiodiplodins by *Lasiodiplodia* sp. 318# (Table 2) [18,47] and by the unidentified endophytic fungus ZZF36 [36] suggests the possible belonging of these isolates to the species *Lasiodiplodia theobromae* and, for this reason, they were included in this review.

The biosynthetic pathways of lasiodiplodin and its 5-hydroxylate derivative (**64**) have been investigated by the administration of ^13^C-labeled acetates to *L. theobromae* [89].

A variety of biological properties have been attributed to lasiodiplodins including antileukemic, antimicrobial activities, and the inhibition of prostaglandin biosynthesis [83]. In particular, lasiodiplodin exhibited antibacterial activity against *S. aureus* and methicillin-resistant *S. aureus* with the respective MIC values of 64 and 128 mg mL^−1^ [41]. Among the hydroxylated analogues [i.e., (3*R*,4*S*)-4-hydroxy-lasiodiplodin (**57**), (3*R*,5*S*)-5-hydroxy-lasiodiplodin (**58**), (3*R*,5*R*)-5-hydroxy-lasiodiplodin (**59**), (3*R*,6*S*)-6-hydroxy-lasiodiplodin (**60**), (3*R*,5*R*)-5-hydroxy-de-*O*-methyl-lasiodiplodin (**64**), (3*R*,6*R*)-6-hydroxy-de-*O*-methyl-lasiodiplodin (**65**), and (3*R*)-5-oxo-lasiodiplodin (**66**)], **57** is the most active in potato microtuber induction tests, showing activity at 10^−3^–10^−4^ M. The presence of an oxydryl group might reduce the antimicrobial activity, and their position and stereochemistry might be involved in the potato microtuber formation [32,33,34,35]. (3*R*)-De-*O*-methyl-lasiodiplodin (**62**) exhibits a good activity against *Trypanosoma brucei* with a minimum inhibitory concentration of 22.5 µM [46] and against *S. aureus* ATCC 29213, *S. aureus* ATCC 700699 and *Enterococcus faecium* ATCC 35667 with MIC 25 μg ml^−1^ [51]. (3*R*,4*R*)-4-Hydroxy-de-*O*-methyl-lasiodiplodin (**63**) and (3*R*,9*E*)-9-etheno-de-*O*-methyl-lasiodiplodin (**71**), together with **62**, **65**, and **68**, were isolated from a cytotoxic extract of *L. theobromae*, endophyte from the root tissue of *Mapania kurzii* [45], but no biological tests were conducted on isolated products. The cytotoxic activities of 12 lasiodiplodins, isolated from a mangrove endophytic strain, were evaluated in vitro against human cancer lines THP1, MDA-MB-435, A549, HepG2, and HCT-116. Compounds **77** and **80** exhibited moderate cytotoxic activities [18,47]. 

### 2.8. Melleins

**Figure 9:**
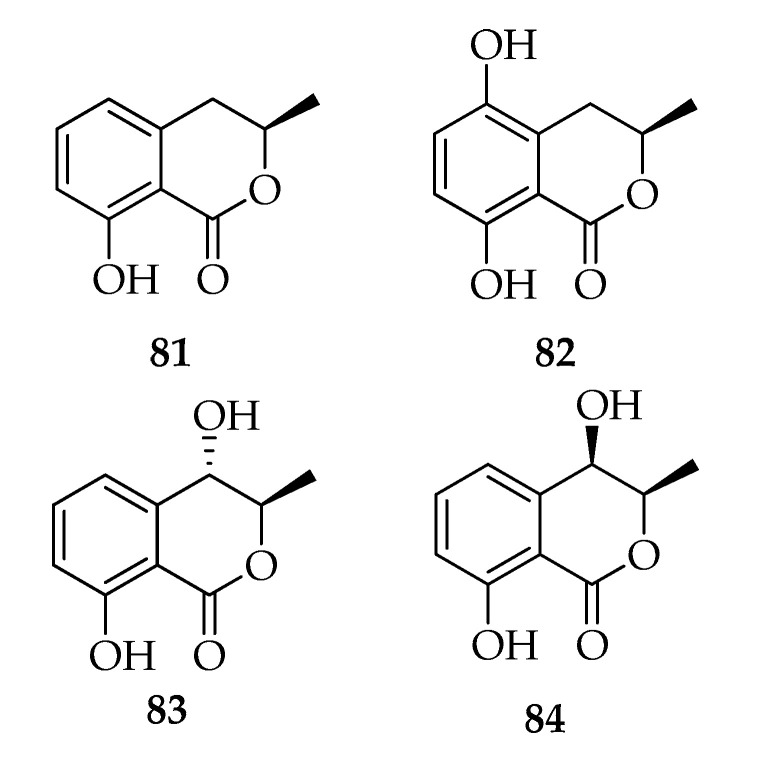
Structures of melleins.

3,4-Dihydroisocoumarins (melleins) are lactonic natural products abundant in microorganisms and higher plants with many biological activities [90] (Figure 9). Some strains of *L. theobromae* are producers of (*-*)-mellein (**81**), (3*R*)-5-hydroxymellein (**82**), (3*R*,4*S*) and (3*R*,4*R*)-4-hydroxymelleins (**83**,**84**), such as strains associated to *Garcina mangostana*, *Viscum coloratum*, *Vitis vinifera*, and *Cocos nucifera* (Table 2). Among the reported strains, the endophytic strain of *G. mangostana* produced the highest variability of melleins [41]. (*-*)-Mellein is produced by most *L. theobromae* strains, while (3*R*,4*R*)-4-hydroxymellein is the only mellein produced by a pathogenic strain of *L. theobromae* isolated from guava [37]. Tests were conducted to investigate the potato tuber formation, and melleins are among the inducing substances produced by *L. theobormae* [30].

### 2.9. Phenyl and Phenol Derivates

**Figure 10:**
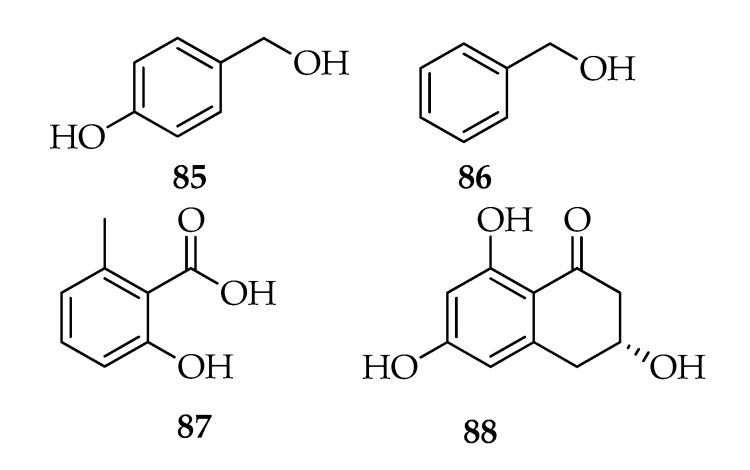
Structures of phenyl and phenol derivatives.

Tyrosol (**85**) and 2-phenylethanol (**86**) are metabolites commonly produced by plants and microorganism through the shikimate biosynthesis pathway, while 6-methylsalicilic acid (**87**) and scytalone (**88**) are polyketides produced by many fungal species (Figure 10). 2-Phenylethanol, a well-known flavor and fragrance substance with a rose-like odor, produced by rose, narcissi, lilies, and jasmine [91] is also produced by fungi [92]. In particular, it is the main product of an endophytic strain of *Lasiodiplodia* sp. isolated from floral part of *Viscum coloratum* [11]. *Lasiodiplodia theobromae* GK-1 produces, in addition to lasiolactone (**54**), 2-phenylethanol [31].

Tyrosol, together with hydroxytyrosol, are the most phenolic compounds present in virgin olive oil; they are responsible for its antioxidant properties [93] and are also produced by many fungal species [94]. Furthermore, tyrosol and phenylethanol are metabolites involved in the quorum sensing for the biofilm development [95]. Tyrosol was identified in two strains of *L. theobromae* pathogen of grapevine, and no effect of temperature was observed on its production [13].

6-Methylsalicylic acid is the precursor of patulin, which is a mycotoxin produced by several *Aspergillus* and *Penicillium* species [96]. It was identified as the product of an endophytic strain of *L. theobromae* PSU-M114 isolated from the leaves of *G. mangostana* [41].

### 2.10. 2-(2-Phenylethyl)chromones

**Figure 11:**
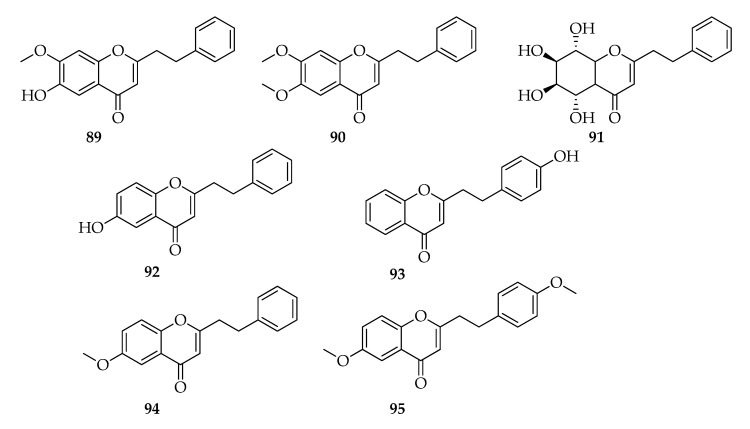
Structures of 2-(2-phenylethyl)chromones.

The endophytic strain of *Botryosphaeria rhodina* A13 was isolated from a 30-year-old *Aquilaria sinensis*, grown in solid medium on sawdust of the host plant with 60% moisture content, and incubated for 38 d in the dark at 27 °C. The ethanolic extract of the culture was submitted to extraction processes using solvents with increasing polarity and to chromatographic purification obtaining seven 2-(2-phenylethyl)chromones identified as: 6-hydroxy-7-methoxy-2-(2-phenylethyl)chromone, 6,7-dimethoxy-2-(2-phenylethyl)chromone, (5*S*,6*R*,7*S*,8*R*)-2-(2-phenylethyl)-5,6,7,8-tetrahydrchromone, 6-hydroxy-2-(2-phenylethyl)chromone, 4-hydroxy-2-(2-phenylethyl)chromone, 6-methoxy-2-phenethyl-4H-chromen-4-one, and 6-methoxy-2-(4-methoxyphenethyl)-4H-chromen-4-one (**89–95**) [50] (Figure 11).

2-(2-Phenylethyl) chromones are an uncommon class of chromones isolated from a few plant species such *Eremophila georgei*, *Bothriochloa ischaemum*, *Imperata cylindrica*, *Cucumis melo,* and *Aquilaria* spp. To this class belong compounds with promising biological activities such as neuroprotective, cytotoxic, acetylcholinesterase inhibitory, antibacterial, and anti-inflammatory [97].

2-(2-Phenylethyl)chromones are among the most abundant constitutes of the agarwood, which is a resinous part of the non-timber Aquilaria tree, which is a highly valuable product for medicine and fragrance purposes [98]. Considering the economic importance of this product and the need to preserve Aquilaria species, some strategies were developed to produce agarwood. Among them, microbial cultures containing *Aspergillus* sp., *Chaetomium* sp., *Fusarium* sp., *Lasiodiplodia* sp., *Penicillium* sp., and *Xylaria* sp. were inoculated in wood of *A. sinensis* [98].

### 2.11. Phytohormones

Phytohormones are commonly associated with plants; they also are present in a wide variety of organisms, including fungi [99,100]. Considering that the physiologic effects of these substances are dose-dependent, fungi with different lifestyles, from necrotrophs to symbionts, are able to produce phytohormones. For instances, 3-indolacetic acid (**17**) and giberellines (GAs) are often produced by species inhabiting rhizosphere, such as *Colletotrichum* sp. from *Artemisia annua* [101] or *Talaromyces verruculosus* from roots of *Potentilla fulgens* [102], while *Fusarium* (=*Gibberella*) *fujikuroi* inducing disease symptoms in rice plants through the production of giberellines [103].

Salicylic acid (**96**) is indirectly responsible for the Systemic Acquired Resistance (SAR) in plants [104]. To date, few fungal species are showed to produce salicylic acid, such as *Moniliophthora perniciosa* and *Oudemansiella mucida* [105]. 

Some fungi produce many phytohormones, including white-rot fungus *Lentinus tigrinus* and the brown-rot fungus *Laetiporus sulphureus* produce abscisic acid (**97**), gibberellic acid (**98**), and cytokinin when grown in the medium of olive oil mill waste [106]. 3-Indolacetic acid, gibberellic acid, abscisic acid, and jasmonic acid (**22**) in several combinations were observed by a pool of unidentified endophytic fungi recovered from five plants used in Indian ethnomedicine [107].

**Figure 12:**
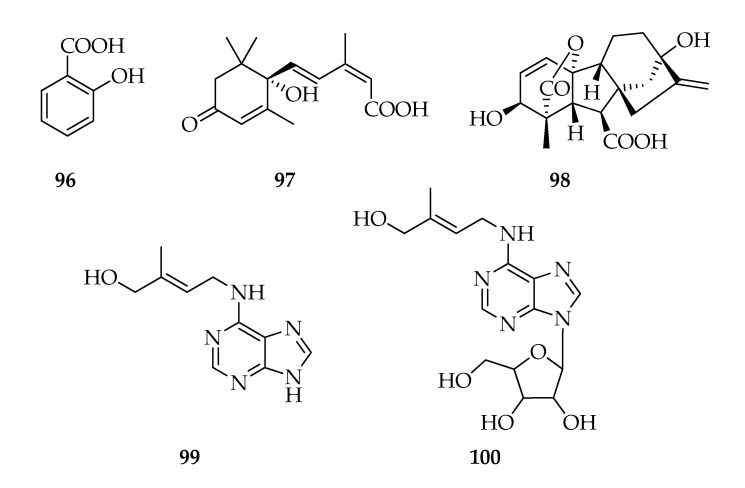
Structures of phytohormones.

Several *Lasiodiplodia theobromae* strains produce phytohormones. In particular, jasmonic acid, analogues, and indole derivatives, such as auxine, are reported in Section 2.5. Contrary to what observed for jasmonates and 3-indolecarboxylic acid, which are often found in cultures of *L. theobromae*, salicylic acid, abscisic acid, giberellines, and cytokinines were only identified in 2334, 1517, and 83 strains of *L. theobromae* [44]. In fact, using high-performance liquid chromatography–electrospray tandem mass spectrometry (HPLC–ESI–MS/MS) method, many phytohormones were identified and quantified in fermentation broths of these strains. In particular, salicylic acid, abscisic acid, gibberellic acid, zeatin, and zeatin riboside (**96–100**) were revealed in a concentration range from 0.5040 to 0.0126 µg mL^−1^. Among the examined strains, the strain 2334 produces the higher amount of **96**, but the lower amount of **100** [44] (Figure 12).

### 2.12. Preussomerins

The preussomerins are a family of about 20 natural compounds identified for the first time in 1990 from the coprophilous fungus *Preussia isomera* [108] and subsequently from other fungal species, including *Sporormiella vexans*, and *Edenia gomezpompae*. Preussomerins possess two naphthalene units linked by three oxygen atoms, generating a bis-spiroacetal system. This remarkable head-to-tail trioxabicyclo[3.3.1]nonane nucleus represents a unique natural product unit and a challenging target for total synthesis [109].

**Figure 13:**
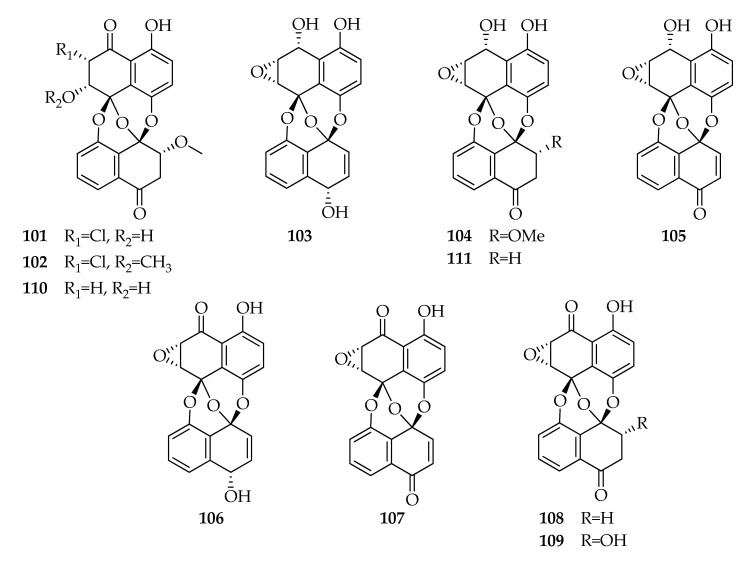
Structures of preussomerins.

Preussomerins (**101–111**) were identified as a product in only one strain of *L. theobromae*. The strain ZJ-HQ_1_ was collected from healthy leaves of the marine mangrove *Acanthus ilicifolius* and cultured on rice solid-substrate medium. An organic extract of fermentation broth of the fungus showed moderate cytotoxicity against human lung cancer cell line and was subsequently submitted to chemical investigations, which led to the isolation and identification of compounds reported in Figure 13. Cloropreussomerins A and B (**101**,**102**) and preussomerins A, C, F, G and H (**103**,**104**,**106**–**108**) showed cytotoxicity against five human cell lines [15].

### 2.13. Miscellaneous 

**Figure 14:**
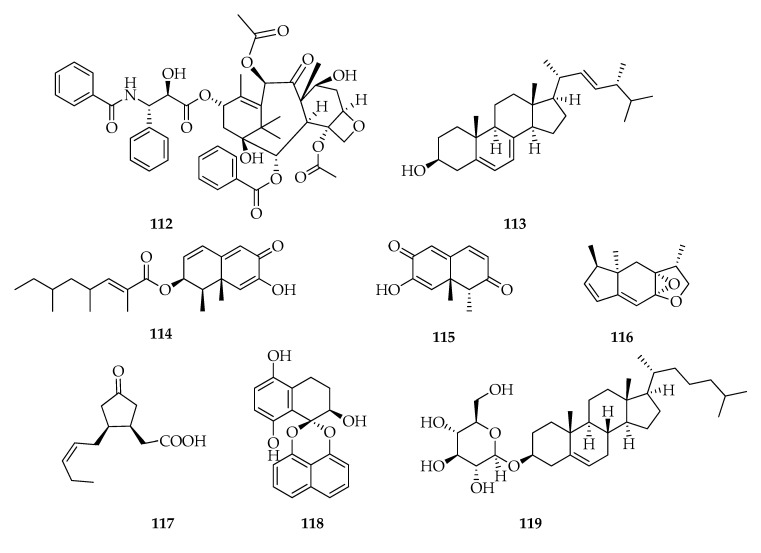
Structures of compounds from the group “miscellaneous”.

Investigations conducted on two endophytic strains of *L. theobromae,* BT155 isolated from *Taxus baccata* and MUBL-BT associated with leaves of the medicinal plant *Morinda citrifolia* showed the production of the antitumor agent named taxol (**112**) [39,43] (Figure 14). Taxol (generic name paclitaxel) is one of the most important natural products in terms of biomedical application and commercial value (i.e., millions dollars per year) [110], which was originally isolated from the bark of Pacific yew, *Taxus brevifolia* in very low concentrations [111], but subsequently, many researchers reported on taxol-producing endophytic fungus. In fact, since 2001, taxol has been isolated from about 20 genera of endophytic fungi, such as *Alternaria*, *Botryosphaeria*, *Botrytis*, *Cladosporium*, F*usarium*, and *Phoma* [112].

*L. theobromae* #009, isolated from guava plants, when cultivated on rice produces ergosterol (**113**), while in liquid medium (i.e., Czapek), it produces *cis*-4-hydroxymellein and a new eremophilane-type sesquiterpene. This metabolite was spectroscopically characterized as 2,4,6-trimethyloct-2-enoic acid, 1,2,6,8a-tetrahydro-7-hydroxy-1,8a-dimethyl-6-oxo-2-naphtalenyl ester (**114**) [37]. Interestingly, **114** is related to botryosphaeridione (**115**), a new dihydronaphthalene-2,6-dione, which is produced in liquid culture by the strain PSU-M35 isolated from *G. mandostana* [41]. New hexahydroindenofuran and cyclopentanone, named respectively botryosphaerihydrofuran (**116**) and botryosphaerinone (**117**), were isolated from the same culture [41], while endophyte strain VP 01 isolated from fresh healthy leaves of *Vitex pinnata* was shown to produced cladospirone B (**118**), which is a member of the spirobisnaphthalene family [113] (Figure 14). Cladospirone B exhibits good activity against *Trypanosoma brucei* with a minimum inhibitory concentration of 17.8 µM [49].

From *Saraca asoca* endophytic strains of *L. theobromae,* a novel steroidal saponin, named cholestanol glucoside (**119**) was isolated and characterized. Its cytotoxic activity was in vitro assessed against six human cancer cell lines A549, PC3, HepG2, U251, MCF7, and OVCAR3, and among them, A549 is the most sensitive cell line [47]. In addition, the antioxidant activity of **119** was tested using different techniques. This compound could efficiently scavenge hydrogen peroxide (IC_50_ value of 7.2 µM) and hydroxyl radicals (IC_50_ value of 3.6 µM) [114]. Moreover, the synergistic effect of **119** and **112** has been assessed against the cervical cancer cell line, HeLa [115].

## 3. Fatty acids

Fatty acids are commonly used in studies of microbial ecology to determine the biomass and structures of microbial communities [116,117], but researchers have found that a large number of fatty acids also have diverse biological activities and functions [118,119,120]. Fatty acids represent the starting material for many secondary metabolites involved in fungal virulence, such as jasmonates [121], and they are also energy-rich compounds and a source of acetyl CoA for polyketide-type metabolites. Fungal lipases are responsible for the degradation of cell membranes and storage lipids in order to obtain free fatty acids and, for this reason, they are important enzymes for the pathogenicity of several fungi [122]. A variety of free fatty acids and their esters was identified in cultures of botryosphaeriaceous fungi, including *L.* *theobromae* [22,72], *Neofusicoccum parvum*, and *N. vitifusiforme* [123]. The investigations conducted on cultures of *L. theobromae* strains isolated in California, Mexico [22], and Cuba [72] revealed a high production and a wide variety of fatty acids and their esters with significant effects on tobacco plants (Table 3). It is proposed that fatty acids be considered plant growth regulators due to their ability to affect tobacco germination and early growth [22]. The detection of octadecenoid acids (e.g., linoleic and linolenic acids) is of great interest because they are precursors of the plant hormone jasmonic acid, which is capable of inducing phytotoxic effects [19,29,72,73]. Hence, octadecenoid intermediates are involved in the fungal virulence because they may participate in the signaling pathway in response to pathogen attack and in plant colonization [121]. Uranga and co-workers [22] also tested the effect of the carbon source on fatty acids production showing the capacity of *L. theobromae* to change its fatty acid metabolism according to the nutrient availability, leading to relevant implications in plant pathogenicity [22].

## 4. Effect of Growth Conditions on Low Molecular Weight Compounds Production

The relevance of studying fungal metabolomic profiles is related to the important biological roles of low molecular weight compounds, such as virulence factors, chemical defense agents, and chemical signals for the communication with other organisms [20,124]. This suggests that secondary metabolites are produced by fungi as physiological responses to multiple biotic and abiotic stimuli, which may affect the expression of biosynthetic gene clusters. In fact, the increasing number of studies on fungal genome sequences showed that the capability of fungi to produce secondary metabolites has been largely underestimated, because many of the fungal secondary metabolite biosynthetic gene clusters are silent under standard cultivation conditions. A large number of genes discovered by bioinformatic analyses based on polyketide synthase (PKS) and non-ribosomal peptide synthetase (NRPS) genes, putatively involved in secondary metabolites biosynthesis, are much higher than estimated, even in fungal species extensively studied for the production of secondary metabolites [125,126]. 

From the observation of fungal genome sequences and considering that the secondary metabolites production might be strain-specific and environment-dependent, investigations by altering process parameters during the fungi cultivation (e.g., temperature and medium composition) might be useful in view of a complete elucidation of fungal metabolome. There is a general lack of information regarding the effect of growth conditions on secondary metabolites produced by fungi, but this crucial issue has been recently addressed for *L. theobromae*. In a recent research activity conducted on four strains of *L. theobromae,* different metabolomic profiles have been reported according to the strain and growth temperature. In addition to several known secondary metabolites, such as JA (**22**) and 3-ICA (**18**), in these studies [13,19], scytalone (**88**) was identified in *Lasiodiplodia* species for the first time, and 3-*epi*-botryodiplodin was also reported for the first time as a natural product. Furthermore, biological investigations showed that the different metabolites production might have implication in fungal virulence and pathogenicity.

The effect of nutrient availability on the production of fatty acids and modified fatty acids by two strains of *L. theobromae* was also investigated, with reference to their importance during the colonization of plants [22]. The findings of Uranga and co-workers [22] show that the variety of compounds produced by *L. theobromae* strains is affected by the carbon source with implication in tobacco physiology related to the fatty acids action as growth regulators during germination and early growth.

An isolate of *B.* *rhodina* (= *L. theobroame*), which is an endophyte of the medicinal plant *Bidens pilosa*, was cultivated in four different culture media both as stationary and as shaken cultures in order to study the production of secondary metabolites. The resulting extracts were subjected to antimicrobial activity and cytotoxicity assays, the most active extract was produced in medium 25 (M25) stationary culture, and four metabolites belonging to depsidones series were identified. These findings confirm that the metabolite production is affected by nutrient availability with implication in the extract activities [42].

The effect of fermentation conditions on *L. theobromae* can be also investigated to enhance the production of compounds with economic importance. This is the case of jasmonates, which are valuable feedstocks and important ingredients in several cosmetic and pharmaceutical preparations [127]. JA is naturally synthesized by plants in very small amounts, and for this reason, the isolation of this compound for industrial purpose is difficult and expensive. The production of JA by fungi in higher amounts than by plants suggested the exploitation of these sources for the industrial production [128]. The optimization of JA production using the fermentation of *L. theobromae* has been an important study conducted testing different strains and altering medium composition [16,129,130,131,132]. 

As documented by Kamal et al. [49], the ideal fermentation condition may be chosen for scaling up the culture of *L. theobromae* to search for compounds with a specific bioactivity (e.g., anti-tripanosomal activity). In fact, the endophyte obtained from *Vites pinnata* was grown in solid rice cultures and liquid Wickerham cultures for 7, 15, and 30 days, after which the metabolomics analysis along with the anti-tripanosomal assays were performed. Based on secondary metabolites production and on the bioassay results, the 30-day rice culture extract exhibited the strongest activity against *Trypanosoma brucei* [49]. 

In general, the optimization of parameters for the maximum production of metabolites of interest is laborious and time consuming because the conventional approach is the one factor at a time method, which involves the alteration of one variable while fixing the others in order to monitor the effect of the altered factor. Interestingly, Valayil and Jayabaskaran [133] used this approach for the optimization of carbon and nitrogen sources [130] for the production of cholestanol glucoside (**119**) in cultures of *L. theobromae* isolated from *Saraca asoca*, and a statistical method (i.e., response surface methodology) to determine the optimum values of trace elements. In fact, although the one factor at time method tends to give more accurate results compared to the response surface methodology, this latter approach is an efficient way for evaluating the effects of multiple factors in a minimum number of trials. Moreover, the same strain was also investigated for modulating the biosynthesis of cholestanol glucoside using oxidative and osmotic stress factors observing an appreciable yield of **119** when *L. theobromae* cultures were subjected to oxidative treatments [134]. 

## 5. Conclusions

In the present review paper, we focus on the available information concerning the extensive literature on secondary metabolites produced by *L. theobromae*, showing that this fungal species produces a high variety of compounds with different chemical and biological proprieties. *L. theobromae* can be also regarded as a producer of high molecular weight compounds (e.g., polysaccharides), which can be the focus of future review projects, considering that no comprehensive review on this topic has been published so far. 

In recent years, the utilization of metabolomics analyses in natural products research provides a very convenient tool to detect known and new compounds produced by *L. thoebromae*. In fact, the metabolomics approach should contribute to an improved knowledge of the linkages between growth conditions, chemical composition, and implication of their occurrence. Further research should be focusing on the exploitation of metabolomics tools for a detailed screening of biochemical profiles of *L. theobromae* strains associated with diverse hosts and lifestyles in order to account for natural variability in chemical composition. Furthermore, new data on the observed valuable bioactivities of compounds might be useful for the economically viable development of high-quality commercial products from *L. theobromae*. In fact, these strategies are promising for the implementation of drug discovery programs.

## Figures and Tables

**Table 1:** List of secondary metabolites produced by *Lasiodiplodia theobromae* gathered from the literature.

Code	Name	Formula	Nominal Mass (U)
**Cyclohexenes and Cyclohexenones**			
**1**	Theobroxide	C_7_H_10_O_3_	142
**2**	(4*S*,5*S*)-4,5-Dihydroxy-2-methylcyclohex-2-enone	C_7_H_10_O_3_	142
**3**	(4*S*,5*S*)-4,5-Dihydroxy-3-methylcyclohex-2-enone	C_7_H_10_O_3_	142
**4**	(4*R*,5*R*)-4,5-Dihydroxy-3-methylcyclohex-2-enone	C_7_H_10_O_3_	142
**5**	(3aS,4*R*,5*S*,7a*R*)-4,5-Dihydroxy-7-methyl-3a,4,5,7a-tetrahydrobenzo[1,3]dioxol-2-one	C_8_H_10_O_5_	186
**6**	(3aR,4S,5R,7aS)-4,5-Dihydroxy-6-methyl-3a,4,5,7a-tetrahydrobenzo[d][1,3]dioxol-2-one	C_8_H_10_O_5_	186
**Depsidones**			
**7**	Botryorhodine A	C_16_H_12_O_6_	300
**8**	Botryorhodine B	C_17_H_14_O_6_	314
**9**	Botryorhodine C	C_17_H_16_O_6_	316
**10**	Botryorhodine D	C_16_H_14_O_6_	302
**11**	Botryorhodine I	C_16_H_14_O_7_	318
**12**	1H-Dibenzo[b,e][1,4]dioxepin-11-one,3,8-dihydroxy-4-(methoxymethyl)-1,6-dimethyl	C_17_H_16_O_6_	316
**13**	Simplicildone A	C_18_H_18_O_6_	330
**Diketopiperazines**			
**14**	*Cyclo*-(Trp-Ala)	C_14_H_15_N_3_O_2_	257
**15**	*Cyclo*-(Phe-Pro)	C_14_H_16_N_2_O_2_	244
**16**	*Cyclo*-(Leu-Pro)	C_11_H_18_N_2_O_2_	210
**Indoles**			
**17**	3-Indolacetic acid (3-IAA)	C_10_H_9_NO_2_	175
**18**	3-Indolcarboxylic acid (3-ICA)	C_9_H_7_NO_2_	161
**19**	3-Indolcarbaldehyde	C_9_H_7_NO	145
**20**	3-Indolpropionic acid (3-IPA)	C_11_H_11_NO_2_	189
**21**	3-Indolbutyric acid (3-IBA)	C_12_H_13_NO_2_	203
**Jasmonates**			
**22**	Jasmonic acid (JA)	C_12_H_18_O_3_	210
**23**	Methyl jasmonate	C_13_H_20_O_3_	224
**24**	(11*S*)-11-Hydroxy-jasmonic acid	C_12_H_18_O_4_	226
**25**	(11*R*)- 11-Hydroxy-jasmonic acid	C_12_H_18_O_4_	226
**26**	8-Hydroxy-jasmonic acid	C_12_H_18_O_4_	226
**27**	12-Hydroxy-jasmonic acid	C_12_H_18_O_4_	226
**28**	3-Oxo-2-(1-hydroxy-2*Z*-pentenyl)cyclopent-1-yl-butyric acid	C_14_H_22_O_4_	254
**29**	3-Oxo-2-(4-hydroxy-2*Z*-pentenyl)cyclopent-1-yl-butyric acid	C_14_H_22_O_4_	254
**30**	JA–Glycine	C_14_H_21_NO_4_	267
**31**	JA–Isoleucine	C_18_H_29_NO_4_	323
**32**	JA–Serine	C_15_H_23_NO_5_	297
**33**	JA–Threonine	C_16_H_26_NO_5_	311
**34**	(+)-7-*iso*-Jasmonic acid	C_12_H_18_O_3_	210
**35**	Ethyl (+)-7-*iso*-jasmonate	C_14_H_22_O_3_	238
**36**	(+)-9,10-Dihydro-7-*iso*-jasmonic acid	C_12_H_20_O_3_	212
**37**	(+)-4,5-Didehydro-7-*iso*-jasmonic acid	C_12_H_16_O_3_	208
**38**	(+)-11,12-Didehydro-7-*iso*-jasmonic acid	C_12_H_16_O_3_	208
**39**	(1*R*,2*S*)-[3-Oxo-2-(2*Z*)-pentenyl)-cyclopentyl]propanoic acid	C_13_H_20_O_3_	224
**40**	(1*S*,2*S*)-[3-Oxo-2-(2*Z*)-pentenyl)-cyclopentyl]butanoic acid	C_14_H_22_O_3_	238
**41**	(+)-Cucurbic acid	C_12_H_20_O_3_	212
**Lactones and Analogues**			
**42**	(3*R*,4*S*)-(-)-Botryodiplodin	C_7_H_12_O_3_	144
**43**	(3*S*,4*S*)-3-*epi*-Botryodiplodin	C_7_H_12_O_3_	144
**44**	(3*S*,4*S*)-4-Acetyl-3-methyldihydrofuran-2(3H)-one	C_7_H_10_O_3_	142
**45**	(3*R*,4*S*)-4-Acetyl-3-methyldihydrofuran-2(3H)-one	C_7_H_10_O_3_	142
**46**	(3*S*,4*R*,5*R*)-4-Hydroxymethyl-3,5-dimethyldihydro-2-furanone	C_7_H_12_O_3_	144
**47**	Botryosphaerilactone A	C_14_H_24_O_5_	272
**48**	Botryosphaerilactone B	C_15_H_24_O_5_	284
**49**	Botryosphaerilactone C	C_14_H_24_O_5_	272
**50**	Lasiolactol A	C_14_H_26_O_5_	274
**51**	Lasiolactol B	C_14_H_26_O_5_	274
**52**	(3*S*,4*R*)-3-Carboxy-2-methylene-heptan-4-olide	C_9_H_12_O_4_	184
**53**	Decumbic acid	C_9_H_12_O_4_	184
**54**	Lasiolactone/(*R*)-(-)-2-Octeno-δ-lactone	C_8_H_12_O_2_	140
**55**	Tetrahydro-4-hydroxy-6-propylpyran-2-one	C_8_H_14_O_3_	158
**Lasiodiplodins**			
**56**	(3*R*)-Lasiodiplodin	C_17_H_24_O_4_	292
**57**	(3*R*,4*S*)-4-Hydroxy-lasiodiplodin	C_17_H_24_O_5_	308
**58**	(3*R*,5*S*)-5-Hydroxy-lasiodiplodin	C_17_H_24_O_5_	308
**59**	(3*R*,5*R*)-5-Hydroxy-lasiodiplodin	C_17_H_24_O_5_	308
**60**	(3*R*,6*S*)-6-Hydroxy-lasiodiplodin	C_17_H_24_O_5_	308
**61**	Botryosphaeriodiplodin	C_17_H_24_O_5_	308
**62**	(3*R*)-De-*O*-methyl-lasiodiplodin	C_16_H_22_O_4_	278
**63**	(3*R*,4*R*)-4-Hydroxy-de-*O*-methyl-lasiodiplodin	C_16_H_22_O_5_	294
**64**	(3*R*,5*R*)-5-Hydroxy-de-*O*-methyl-lasiodiplodin	C_16_H_22_O_5_	294
**65**	(3*R*,6*R*)-6-Hydroxy-de-*O*-methyl-lasiodiplodin	C_16_H_22_O_5_	294
**66**	(3*R*)-5-Oxo-Lasiodiplodin	C_17_H_22_O_5_	306
**67**	(3*R*)-7-Oxo-Lasiodiplodin	C_17_H_22_O_5_	306
**68**	(3*R*)-6-Oxo-de-*O*-methyl-lasiodiplodin	C_16_H_20_O_5_	292
**69**	(3*R*,5*E*)-5-Etheno-lasiodiplodin	C_17_H_22_O_4_	290
**70**	(3R,9*E*)-9-Etheno-lasiodiplodin	C_17_H_22_O_4_	290
**71**	(3*R*,9*E*)-9-Etheno-de-O-methyl-lasiodiplodin	C_16_H_20_O_4_	276
**72**	(*R*)-14-Methoxy-3-methyl-3,4,5,6,7,8,9,10-octahydro-1H-benzo[c][1]oxacyclododecine-1,11,12-trione	C_17_H_22_O_5_	306
**73**	Lasiodiplactone	C_24_H_34_O_5_	402
**74**	*epi*-8,9-Dihydrogreensporone C	C_19_H_26_O_5_	334
**75**	(3*R*)-Nordinone	C_18_H_24_O_5_	320
**76**	(*R*)-Zearalenone	C_18_H_26_O_4_	306
**77**	Ethyl-2,4-dihydroxy-6-(8’-hydroxynonyl)benzoate	C_18_H_28_O_5_	324
**78**	Isobutyl-2,4-dihydroxy-6-(8’-hydroxynonyl)benzoate	C_20_H_32_O_5_	352
**79**	Ethyl-2,4-dihydroxy-6-(8’-oxononyl)benzoate	C_18_H_26_O_5_	322
**80**	Ethyl-2,4-dihydroxy-6-nonylbenzoate	C_18_H_28_O_4_	308
**Melleins**			
**81**	(-)-Mellein	C_10_H_10_O_3_	178
**82**	(-)-(3*R*)-5-Hydroxymellein	C_10_H_10_O_4_	194
**83**	(-)-(3*R*,4*S*)-(*trans*)-4-Hydroxymellein	C_10_H_10_O_4_	194
**84**	(-)-(3*R*,4*R*)-(*cis*)-4-Hydroxymellein	C_10_H_10_O_4_	194
**Phenyl and Phenol derivates**			
**85**	Tyrosol	C_7_H_8_O_2_	124
**86**	2-Phenylethanol	C_7_H_8_O	108
**87**	6-Methylsalicylic acid	C_8_H_8_O_3_	152
**88**	Scytalone	C_10_H_10_O_4_	194
**2-(2-Phenylethyl)chromones**			
**89**	6-Hydroxy-7-methoxy-2-(2-phenylethyl)chromone	C_18_H_16_O_4_	296
**90**	6,7-Dimethoxy-2-(2-phenylethyl)chromone	C_19_H_18_O_4_	310
**91**	(5*S*,6*R*,7*S*,8*R*)-2-(2-Phenylethyl)-5,6,7,8-tetrahydrchromone	C_17_H_20_O_6_	320
**92**	6-Hydroxy-2-(2-phenylethyl)chromone	C_17_H_14_O_3_	266
**93**	4-Hydroxy-2-(2-phenylethyl)chromone	C_17_H_14_O_3_	266
**94**	6-Methoxy-2-phenethyl-4H-chromen-4-one	C_18_H_16_O_3_	280
**95**	6-Methoxy-2-(4-methoxyphenethyl)-4H-chromen-4-one	C_19_H_18_O_4_	310
**Phytohormones**			
**96**	Salicylic acid	C_7_H_6_O_3_	138
**97**	Abscisic acid	C_15_H_20_O_4_	264
**98**	Giberellic acid (GA3)	C_19_H_22_O_6_	346
**99**	Zeatin	C_10_H_13_N_5_O	219
**100**	Zeatin riboside	C_15_H_21_N_5_O_5_	351
**Preussomerins**			
**101**	Chloropreussomerin A	C_21_H_15_ClO_8_	430
**102**	Chloropreussomerin B	C_22_H_17_ClO_8_	444
**103**	Preussomerin A	C_20_H_14_O_7_	366
**104**	Preussomerin C	C_21_H_16_O_8_	396
**105**	Preussomerin D	C_20_H_12_O_7_	364
**106**	Preussomerin F	C_20_H_12_O_7_	364
**107**	Preussomerin G	C_20_H_10_O_7_	362
**108**	Preussomerin H	C_20_H_12_O_7_	364
**109**	Preussomerin K	C_20_H_12_O_8_	380
**110**	Preussomerin M	C_21_H_16_O_8_	396
**111**	Ymf 1029	C_20_H_14_O_7_	366
**Miscellaneous**			
**112**	Taxol	C_47_H_51_NO_14_	853
**113**	Ergosterol	C_28_H_44_O	396
**114**	2,4,6-Trimethyloct-2-enoic acid 1,2,6,8a-tetrahydro-7-hydroxy-1,8a-dimethyl-6-oxo-2-naphtalenyl ester,	C_23_H_32_O_4_	372
**115**	Botryosphaeridione	C_12_H_12_O_3_	204
**116**	Botryosphaerihydrofuran	C_14_H_18_O_2_	218
**117**	Botryosphaerinone	C_12_H_18_O_3_	210
**118**	Cladospirone B	C_20_H_16_O_5_	336
**119**	Cholestanol glucoside	C_33_H_56_O_6_	548

**Table 2:** Occurrence of secondary metabolites in *Lasiodiplodia theobromae* strains. Strains are listed according to the investigation date. Fungal lifestyles were reported when available in the original papers.

Strain	Source (Lifestyle)	Growth Conditions	Identified Compounds *	Bioactivity	Ref.
Cellulolytic strain	-	PDB shaken, 8 d, 30 °C	**42**	Antibacterial	[24]
-	-	Czapek medium	**18,19,22,56,62,81,84**	-	[25]
D 7/2	*Citrus sinensis*	Medium (sucrose, soya flour, corn steep liquor, mineral salts), 7 d, 30 °C	**24–29,34–41**	-	[26,27,28]
-	-	Czapek medium (0.1% yeast extract), 15 d, 26 °C	**22**	Phytotoxic	[29]
IFO 31059	-	Potato–sucrose medium, 30 d, 23 °C	**1,22,23,81**	Potato microtuber induction	[30]
GK-1	*Cocos nucifera* (endophyte)	Potato dextrose agar (PDA), 15 d, 25 °C	**54,81,86**	-	[31]
IFO 31059	-	Potato–sucrose medium (2%), 35 d, 23 °C	**58,59,66**	Potato microtuber induction	[32]
IFO 31059 (mycelium)	-	Potato–sucrose medium (3%), 35 d, 25 °C	**57,64,65,77,78**	Potato microtuber induction	[33,34]
-	*Mangifera indica* (pathogen)	Surface-sterilized bananas, 3 d, 25 °C	**52,53**	Phytotoxic	[23]
		Potato–glucose, 21 d, 25 °C	**52**		
Shimokita 2	*Mangifera indica*	Potato–sucrose medium (3% sucrose) 21 d, 25 °C	**60**	Potato microtuber induction	[35]
ZZF36	*Sargassum* sp. (endophyte)	-	**56,62,64,68,70**	Antimicrobial	[36]
-	*Psidium guajava* (pathogen)	Rice, 32 d, room temperature	**113**	-	[37]
		Czapek, 40 d, room temperature	**84,114**		
OCS71	-	Potato dextrose broth (PDB, 2%), 21 d, 25 °C	**2,5**	Potato microtuber induction	[38]
BT 115	*Taxus baccata* (endophyte)	-	**112**		[39]
OCS71	-	PDB (2%), 14 d, 25 °C	**1,3,4,6**	-	[40]
PSU-M114	*Garcinia mangostana* (endophyte)	PDB, 21 d, room temperature	**54–56,58,59,61,81–84,87,117**	Antibacterial	[41]
PSU-M35	*Garcinia mangostana* (endophyte)	PDB, 21 d, room temperature	**44,46–49,115,116**	Antibacterial	[41]
*-*	*Bidens Pilosa* (endophyte)	M25, 21 d, 23 °C	**7–10,18**	Antimicrobial, antiproliferative, cytotoxic	[42]
-	*Morinda citrifolia* (endophyte)	MID with soytone (1 g), 22 d	**112**	Cytotoxic	[43]
2334	*Citrus sinensis*	Medium (sucrose, mineral salts and yeast extract), 10 d, 30 °C	**17,20–22,30–33,96–100**	-	[44]
1517	*Citrus sinensis*	Medium (sucrose, mineral salts and yeast extract), 10 d, 30 °C	**17,20–22,30–33,96–100**	-	[44]
83	Brazilian wood	Medium (Sucrose, mineral salts and yeast extract), 10 d, 30 °C	**17,20–22,30–33,96–100**	-	[44]
-	*Mapania kurzii* (endophyte)	PDA	**62,63,65,68,71**		[45]
ZJ-HQ1	*Acanthus ilicifolius*	Rice solid-substrate medium+artificial sea salt solution (3%), 28 d, room temperature	**101–111**	Cytotoxic, Antibacterial	[15]
UCD256Ma	*Vitis vinifera*	5% glucose, 20 d, 25 °C	**81**, fatty acids (Table 3)	Tobacco seed germination	[22]
MXL28	*Vitis vinifera*	Oatmeal powder, 60 d, room temperature	**81**, fatty acids (Table 3)	Tobacco seed germination	[22]
-	*Saraca asoca* (endophyte)	M1D broth, 3 d, 25 °C	**119**	Cytotoxic activity against human cancer lines, antioxidant activity	[46]
318#	*Excoecaria* *agallocha* (endophyte)	Rice solid-substrate medium, 28 d, 28 °C	**56,58,59,62,67,69,72,74, 76,77,79,80**	Cytotoxic activity against human cancer lines	[18,47]
ZJ-HQ1	*Acanthus ilicifolius* (endophyte)	Rice solid-substrate medium+artificial sea salt solution (3%), 28 d, room temperature	**73**	Anti-inflammatory	[17]
SNFF	*Solanum nigrum* (endophyte)	Liquid malt extract medium, 28 d, 20 °C	**15,16,19**	-	[48]
VP 01	*Vitex pinnata* (endophyte)	Rice solid medium, 30 d, room temperature	**62,81,118**	Anti-trypanosomal	[49]
A13	*Aquilaria sinensis* (endophyte)	Saw dust of host plant with 60% moisture content, 38 d, 27 °C	**89–95**	-	[50]
CAA019	*Cocos nucifera* (pathogen)	Czapek amended with cornmeal, 21 d, 25 °C	**18,22,56**	Phytotoxic, cytotoxic	[19]
		Czapek amended with cornmeal, 21 d, 37 °C	**18,22,44–47**		
CBS339.90	Human (pathogen)	Czapek amended with cornmeal, 21 d, 25 °C	**18,22,83,84,88**	Phytotoxic, cytotoxic	[19]
		Czapek amended with cornmeal, 21 d, 37 °C	**14,18,44–47,83,84**		
LA-SOL3	*Vitis vinifera* (pathogen)	Czapek amended with cornmeal, 21 d, 25 °C	**18,22,45,46,50,51,85**	Phytotoxic, cytotoxic	[13]
		Czapek amended with cornmeal, 21 d, 37 °C	**18,42–46,50,51,84,85**		
LA-SV1	*Vitis vinifera* (pathogen)	Czapek amended with cornmeal, 21 d, 25 °C	**18,22,45,46,50,51,81,84**	Phytotoxic, cytotoxic	[13]
		Czapek amended with cornmeal, 21 d, 37 °C	**18,42,43,45,46,50,51,80,85**		
M4.2-2	Mangrove sediment	Rice medium, 25 d, room temperature	**7,8,10–13,62,75,81**	Antibacterial, cytotoxic	[51]

* For definition of codes, see Table 1.

**Table 3:** Fatty acids and esters of fatty acids produced by *Lasiodiplodia theobromae* strains.

Fatty Acids and Their Esters							
Strain	*L. theobromae* UCD256Ma [22]				*L. theobromae* MXL28 [22]	*L. theobromae* CBS 122127 [72]	*L. theobromae* CBS 2334 [72]
Growth Condition	Oatmeal Powder, 60d, Room Temperature	5% Glucose, 20 d, 25 °C	5% Oil, 20 d, 25 °C	5% Oil + 5% Glucose, 20 d, 25 °C	Oatmeal Powder, 60 d, Room Temperature	Czapek-Dox Medium, 10–12 d 27 °C (Mycelium)	Czapek-Dox Medium, 10–12 d, 27 °C (Mycelium)
Hexadecenoic acid (C16:1n7)						+	+
Methyl hexadecanoate (C16:0 ME)	+		+		+		
Ethyl hexadecanoate (C16:0 EE)	+	+	+	+	+		
Hexadecanoate, 2-methylpropyl ester	+				+		
Octadecanoic acid (C18:0)						+	
9-Octadecenoic acid (*Z*) (C18:1n9)						+	
9-Octadecenoate (*Z*)- methyl ester (C18:1n9 ME)	+			+	+		
Octadecanoate ethyl ester (C18:0 EE)	+		+	+	+		
9-Octadecenoate (*Z*), ethyl ester (C18:1n9 EE)	+		+	+	+		
9-Octadecenoate (*E*) ethyl ester (C18:1n9 EE)	+		+	+			
9,12-Octadecadienoic acid (*Z,Z*) (C18:1n9)						+	+
9,12-Octadecadienoate (*Z,Z*)-, methyl ester (C18:1n9 ME)	+		+	+	+		
9,12-Octadecadienoate (*Z,Z*) ethyl Ester (C18:1n9 EE)	+		+	+	+		
9,12,15-Octadecatrienoate (*Z,Z,Z*)-ethyl ester) (C18:3n3 EE)	+		+	+	+		
Eicosanoic acid (C20:0)						+	

+ presence in culture extract.
